# MiR-16 regulates the pro-tumorigenic potential of lung fibroblasts through the inhibition of HGF production in an FGFR-1- and MEK1-dependent manner

**DOI:** 10.1186/s13045-018-0594-4

**Published:** 2018-03-20

**Authors:** Francesca Andriani, Maria Teresa Majorini, Miguel Mano, Elena Landoni, Rosalba Miceli, Federica Facchinetti, Mavis Mensah, Enrico Fontanella, Matteo Dugo, Mauro Giacca, Ugo Pastorino, Gabriella Sozzi, Domenico Delia, Luca Roz, Daniele Lecis

**Affiliations:** 1 Tumor Genomics Unit, Department of Experimental Oncology and Molecular Medicine, Fondazione IRCCS Istituto Nazionale dei Tumori, Milan, Italy; 2 Molecular Mechanisms of Cell Cycle Control Unit, Department of Experimental Oncology and Molecular Medicine, Fondazione IRCCS Istituto Nazionale dei Tumori, Milan, Italy; 3 Molecular Medicine Laboratory, International Centre for Genetic Engineering and Biotechnology (ICGEB), Trieste, Italy; 4 Unit of Medical Statistics, Biometry, and Bioinformatics Fondazione IRCCS Istituto Nazionale dei Tumori, Milan, Italy; 5 Functional Genomics and Bioinformatics Core Facility, Department of Experimental Oncology and Molecular Medicine, Fondazione IRCCS Istituto Nazionale dei Tumori, Milan, Italy; 6 Thoracic Surgery Unit, Department of Surgery, Fondazione IRCCS Istituto Nazionale dei Tumori, Milan, Italy; 7 Present Address: Functional Genomics and RNA-based Therapeutics laboratory, Center for Neuroscience and Cell Biology (CNC), University of Coimbra, 3060-197 Coimbra, Portugal; 8 Present Address: Molecular Immunology Unit, Department of Experimental Oncology and Molecular Medicine, Fondazione IRCCS Istituto Nazionale dei Tumori, Milan, Italy

**Keywords:** Tumor microenvironment, Lung cancer, miR-16, Cancer cell migration, FGFR-1 signaling

## Abstract

**Background:**

Fibroblasts are crucial mediators of tumor-stroma cross-talk through synthesis and remodeling of the extracellular matrix and production of multiple soluble factors. Nonetheless, little is still known about specific determinants of fibroblast pro-tumorigenic activity in lung cancer. Here, we aimed at understanding the role of miRNAs, which are often altered in stromal cells, in reprogramming fibroblasts towards a tumor-supporting phenotype.

**Methods:**

We employed a co-culture-based high-throughput screening to identify specific miRNAs modulating the pro-tumorigenic potential of lung fibroblasts. Multiplex assays and ELISA were instrumental to study the effect of miRNAs on the secretome of both primary and immortalized lung fibroblasts from lung cancer patients and to evaluate plasmatic levels of HGF in heavy smokers. Direct mRNA targeting by miRNAs was investigated through dual-luciferase reporter assay and western blot. Finally, the pro-tumorigenic activity of fibroblasts and their conditioned media was tested by employing in vitro migration experiments and mouse xenografts.

**Results:**

We identified miR-16 as a master regulator of fibroblast secretome and showed that its upregulation reduces HGF secretion by fibroblasts, impairing their capacity to promote cancer cell migration. This effect is due to a pleiotropic activity of miR-16 which prevents HGF expression through direct inhibition of FGFR-1 signaling and targeting of HGF mRNA. Mechanistically, miR-16 targets FGFR-1 downstream mediator MEK1, thus reducing ERK1/2 activation. Consistently, chemical or genetic inhibition of FGFR-1 mimics miR-16 activity and prevents HGF production. Of note, we report that primary fibroblast cell lines derived from lungs of heavy smokers express reduced miR-16 levels compared to those from lungs not exposed to smoke and that HGF concentration in heavy smokers’ plasma correlates with levels of tobacco exposure. Finally, in vivo experiments confirmed that restoration of miR-16 expression in fibroblasts reduced their ability to promote tumor growth and that HGF plays a central role in the pro-tumorigenic activity of fibroblasts.

**Conclusions:**

Overall, these results uncover a central role for miR-16 in regulating HGF production by lung fibroblasts, thus affecting their pro-tumorigenic potential. Correlation between smoking exposure and miR-16 levels could provide novel clues regarding the formation of a tumor-proficient milieu during the early phases of lung cancer development.

**Electronic supplementary material:**

The online version of this article (10.1186/s13045-018-0594-4) contains supplementary material, which is available to authorized users.

## Background

Lung cancer is the leading cause of cancer deaths worldwide due to its high incidence and mortality [1]. Despite recent advances in immunotherapy [2] and targeted therapies [3], at present, less than 10% of non-small cell lung cancer (NSCLC) patients with metastatic disease reach a 5-year survival after diagnosis [4]. Effective treatment still represents a major challenge due to multiple layers of heterogeneity which lead to differential cancer cell aggressiveness and response to therapy [5, 6]. This variability is the result of many cancer cell autonomous mechanisms such as genetic and epigenetic alterations which lead to the perturbation of several pathways [7], but also derives from the influence of tumor microenvironment (TME) [8, 9]. Accordingly, there is growing evidence that development and biological behavior of tumors stem from an extremely complex cross-talk between cancer cells and the surrounding milieu constituted by the extracellular matrix (ECM), soluble factors, immune cell infiltrates [10], and cancer-associated fibroblasts (CAFs) [11]. The understanding of how these extracellular signals influence cancer cells could therefore provide new molecular targets of potential clinical relevance and/or improved prognostic tools [12].

MiRNAs, which are potent regulators of gene expression, add a further level of complexity to this process by controlling multiple pathways within the different cells of the TME and even allowing the cross-talk between distant cells when shuttled by extracellular vesicles [13]. This is the case, for example, of miR-16 which, together with miR-15, was the first miRNA described to be deleted in cancer, specifically in chronic lymphocytic leukemia (CLL) cells [14]. Loss or reduction of these miRNAs promotes several tumorigenic cancer cell features due to the consequent increase of their target genes BCL2 [15], CCND1, and WNT3A [16]. Interestingly, miR-16 also suppresses the fibroblast growth factor-2 (FGF-2)/FGF receptor-1 (FGFR-1) axis [17], and therefore, the loss of this miRNA can eventually enhance cancer cell survival, proliferation, and migration.

The pathological activation of the FGFR-1 receptor is often observed in cancer cells, and it is caused by gene amplification [18] or aberrant stimulation by its cognate ligands, thus contributing to lung cancer patients’ poor prognosis. Activated FGFR-1 in fact hampers the efficacy of therapies directed against epidermal growth factor receptor (EGFR) [19, 20] and cMet [21]. In some tumor types, cells have been shown to acquire resistance to the cMet inhibitor crizotinib through an FGFR-1-dependent compensatory upregulation of HGF [22]. This cytokine stimulates cMet and favors cancer development, angiogenesis, and tumorigenesis through activation of its downstream pathways [23, 24]. Importantly, also, the activation of cMet protects against FGFR-targeted therapy [21], thus suggesting a reciprocal functional interaction between these two receptors. Of note, several findings support the idea that CAFs represent a preferential source of soluble factors which promote cancer aggressiveness [25]. In this context, microenvironment-derived HGF plays a pivotal role in cancer cell resistance to therapy [26, 27], sustains cancer stem cells [28], and promotes invasive growth and dissemination [29].

In our work, we aimed at understanding the mechanisms by which miRNAs affect the pro-tumorigenic features of fibroblasts. After performing a genome-wide functional screening analysis, we focused specifically on miR-16, demonstrating that it regulates HGF secretion by fibroblasts in an FGFR-1-dependent manner. We further identify MEK1 as a novel direct target of miR-16. Our observation that fibroblast-derived HGF promotes cancer cell migration in vitro and tumor development in vivo supports the notion that both cancer and stromal miR-16 levels affect cancer aggressiveness. Importantly, we provide also evidence that tobacco smoking may promote the reduction of miR-16 expression in lung fibroblasts and favor the increase of systemic HGF levels.

## Methods

### Cell lines and reagents

Primary lung fibroblasts (“cancer-associated” (CAFs), obtained from the tumor site, and "adjacent" (AF) or “normal” (NFs), obtained from normal tissue proximal or at least 3 cm from the neoplastic lesion, respectively) were derived from surgical specimens and cultured as already described [8, 30]. All available samples were analyzed, and miRNA expression profiles were deposited in the Gene Expression Omnibus (GEO) Repository (accession number: GSE97545). Human NSCLC cell lines A549 (adenocarcinoma, CCL-185) and Calu-1 (squamous cell carcinoma, HTB-54) cells were purchased from American Type Culture Collection (ATCC, LGC Standards). LT73 primary cell line was derived in our laboratory from a surgical specimen of a male patient with lung adenocarcinoma [8]. Cancer cells were cultured in RPMI 1640 (Lonza) supplemented with 10% fetal calf serum (FCS) [30]; in vivo experiments were performed as described by Bertolini and colleagues [31]. HEK293FT (R700-07) cells were purchased from Invitrogen (Thermo Fisher Scientific, Waltham, MA, USA). All cell lines were routinely tested to exclude the presence of mycoplasma contamination, grown as adherent monolayer, and harvested at controlled density.

For immortalization, primary fibroblasts were transduced with retroviral particles produced as described [32] using the pLX-SP-hTERT vector and cultured in medium plus 1 μg/ml puromycin. The expression levels of the transgene were determined by real-time PCR using TERT TaqMan assay (Thermo Fisher Scientific) and normalized relative to HPRT. Fibroblasts expressing ectopic hTERT were characterized both in vitro (Additional file 1: Figure S1) and in vivo (Additional file 2: Figure S2) to exclude that the immortalization process affected their pro-tumorigenic features. Senescent cells were detected using the Senescence Cells Histochemical Staining Kit (Sigma-Aldrich), according to the manufacturer’s instruction.

Ectopic expression of FGFR-1 and mitogen-activated protein kinase/ERK kinase 1 (MEK1) was obtained by lentiviral particles prepared in HEK293FT cells as described [33] using the pWZL Neo Myr FLAG FGFR1, a gift from William Hahn & Jean Zhao (Addgene #20486) [34], and human Lenti-ORF pLenti-C-Myc-DDK clone of human mitogen-activated protein kinase kinase 1 (MAP2K1, OriGene #RC218460L1), respectively.

For fibroblast transfection, the negative control miRNA #1 (miR-C, #4464058) and hsa-miR-16-5p (miR-16, MC10339) miRNA mimics, the negative control miRNA #1 (miR-C inh, #4464076) and hsa-miR-16-5p (miR-16 inh, MC10339) miRNA inhibitor (Thermo Fisher Scientific), control siRNA (siCtr; 5′-CGUACGCGGAAUACUUCGATT-3′, Eurofins), siFGFR-1 #SI02224677 and #SI02224684, siHGF #SI03046946, siMEK1#6 #SI00300699, and siMEK1#7 #SI02222955 (Qiagen, Hilden, Germany) were used. Transfection of fibroblasts was performed by reverse transfection in 12-well plates, and small RNAs (50 nM final concentration for mimics, 100 nM for miRNA inhibitors) were mixed with Lipofectamine RNAiMAX Transfection Reagent (Thermo Fisher Scientific) to each well. After incubation, 1.5 × 10^5^ fibroblasts were seeded, left to adhere, and incubated for 72 h. Conditioned medium (CM) was then collected for analysis and cancer cell stimulation, and cells were harvested for western blots. Small RNAs targeting essential genes were employed in parallel as internal transfection controls. Transfection efficiency was evaluated by real-time PCR using an hsa-miR-16 probe (391 assay ID, Thermo Fisher). Before stimulation with FGF-2 (ATCC, LGC Standards), fibroblasts were serum-starved for 48 h. Viability was evaluated using Cell TiterGlo reagent (Promega) according to the manufacturer’s instructions. The FGFR-1 inhibitor SU5402 was purchased from Calbiochem.

### Patient and tissue sampling

The selection of cohorts analyzed was approved by the Fondazione IRCCS Istituto Nazionale dei Tumori Ethics Review Board. Written informed consent was obtained from each patient and healthy individual for blood collection. Whole blood samples (5–10 ml) were collected as first blood with spray-coated K_2_EDTA (BD-Becton, Dickinson and Company, Plymouth, UK). Within 2 h, plasma was separated by a first centrifugation step at 1258*g* at 4 °C for 10 min. The supernatant containing plasma was carefully collected avoiding the fraction closest to the lymphocytic ring. Plasma was then centrifuged a second time at 1258*g* at 4 °C for 10 min and collected for further analysis [35].

### Statistical and bioinformatic analyses

In silico prediction of miRNA targets was obtained combining six different algorithms (DIANA microT-CDS [22285563], microrna.org database [18158296], mirDB [18048393], PITA [17893677], RNA22 [16990141], and TargetScan v6.2 [15652477]). Putative mRNA targets predicted by at least five out of six algorithms were selected. The Jaccard Index was calculated for each pair of miRNAs as a measure of similarity between the lists of predicted targets. To identify clusters of miRNAs sharing common targets, we applied hierarchical clustering to the Jaccard Index matrix, with Euclidean distance and average linkage as clustering parameters. Graphs and statistical analysis were performed with GraphPad Prism 5.02.

### Systemic HGF measurement

The analysis of HGF plasmatic levels was performed on a set of 90 healthy heavy smokers enrolled in a lung cancer screening program [36]. Circulating HGF was measured by using commercially available ELISA kits (R&D) according to the manufacturer’s instruction. Duplicate measures were performed for each sample. Protein levels were expressed in OD value measured by Microplate Reader Tecan Infinite® M1000. Raw absorbance values were corrected by exploiting the values of the ELISA standards. Boxplots and Wilcoxon test were used to evaluate the association between HGF and categorical variables; the association between HGF and continuous variables was assessed by means of scatter plots and the calculation of Spearman correlation coefficient. Analyses were carried out using R software, version 3.2.0 (http://www.r-project.org/). The test results were considered statistically significant whenever a two-sided *P* value below 0.05 was achieved.

### High-throughput screening (HTS)

As the large-scale screening experiment was not feasible with primary CAFs due to the limited cell number, fibroblasts (CAFs, AFs, and NFs) from different patients were transduced with retroviral particles to stably express human TERT (hTERT) and immortalize cells (see above). For the high-throughput screening, at day 1, CAF154-hTERT fibroblasts were reverse transfected with a library of human miRNA mimics composed of 988 mature miRNAs arrayed on 96-well plates (875 unique sequences, miRBase v.13.0, miRIDIAN technology, Dharmacon). Briefly, 15 μl miRNA (500 nM) was spotted per well, and a mix of 35 μl Opti-MEM containing RNAiMAX (Thermo Fisher Scientific) was added. After 30 min of incubation, 100 μl of medium containing 8000 fibroblasts was added. A549-green fluorescent protein (GFP) cells (3500 cells/well) were seeded at day 3 (48 h after fibroblast transfection) and co-cultured for further 48 h. The experiment was stopped by fixing the cells with 4% paraformaldehyde, and nuclei were counterstained with Hoechst 33342. Image acquisition was performed using an ImageXpress Micro automated high-content screening fluorescence microscope (Molecular Devices) at a × 4 magnification; a total of four images were acquired per wavelength, well, and replicate, corresponding to ca. 10,000–15,000 cells analyzed per experimental condition and replicate. Image analysis to determine the number of fibroblasts (GFP-negative) and A549 cells (GFP-positive) was performed using the “Multi-Wavelength Cell Scoring” application module implemented in MetaXpress software (Molecular Devices). To score the effect of each miRNA in stimulating or decreasing fibroblast and A549 cell growth, results were normalized per plate, relative to the median of the samples. Screening was performed in duplicate, at the ICGEB High-Throughput Screening Facility (Trieste, Italy). In validation co-culture experiments, the same conditions of the HTS were applied, but plates were incubated in a Cell-IQ SLF instrument (CM Technology Oy, Tampere, Finland) for image acquisition and automated analysis.

### Analysis of conditioned medium (CM) and plasma

For secretome analysis, 1.5 × 10^5^ CAF154-hTERT cells were reverse transfected as described above in 12-well plates and CM was collected 72 h later. Biological triplicates were prepared for each condition, i.e., transfection with miR-C and miR-16. In this study, we quantified simultaneously 64 cytokine/chemokine/growth factor biomarkers by using the Human Cytokine Array/Chemokine Array 64-Plex Discovery Assay, 17 angiogenesis/growth factor biomarkers of the Human Angiogenesis Array and Growth Factor Array, and 13 matrix metalloproteinases/tissue inhibitors of metalloproteinases of the Human MMP and TIMP Panel (Eve Technologies Corp, Calgary, AB, Canada). To quantify HGF in different fibroblast cell lines, serum-free media conditioned for 24 h by 1 × 10^6^ cells were collected and analyzed by ELISA (Human HGF Instant ELISA #BMS2069INST; eBioscience) according to the manufacturer’s instructions.

### Western blot

Western blots were performed as already described [33]. The following primary antibodies were employed: α-cMet #sc-10 (Santacruz); α-Actin #A1978 and α-pERK1/2 #M8159 (Sigma-Aldrich); α-p-cMet #44888G (Life Technologies); α-FGFR-1 #9740, α-MEK1/2 #4694, α-MEK1 #12671, and α-pAKT #9275 (Cell Signaling Technologies); and α-HGF #10679 (Abcam).

### Dual-luciferase reporter assay

For miRNA target validation, the firefly luciferase-expressing pMirTarget plasmids containing the 3′ untranslated region (UTR) of HGF #SC206339, of MEK1 #SC211301, and of FGFR-1 #SC211347 were employed (OriGene). The pMirTarget 3′UTR HGF was mutagenized using QuikChange II XL Site-Directed Mutagenesis Kit (Agilent, Santa Clara, CA) to delete the putative binding site for miR-16. Target sequences are shown in Additional file 3: Figure S3. The HEK293T cells were seeded (5 × 10^5^ cells/well) in 96-well plates and left to adhere overnight. Then, miRNA transfection was performed using Lipofectamine 2000 reagent (100 nM final miRNA concentration) and pMirTarget plasmid (0.1 μg/well), together with the *Renilla* luciferase-expressing pGL4.74 plasmid (0.01 μg/well). After 24 h, both the firefly and *Renilla* luciferase activities were measured by dual-luciferase reporter assay (Promega) according to the manufacturer’s instructions.

### Migration

Cell motility was studied in wound-healing experiments by seeding 6 × 10^4^ A549 cells in each chamber of a culture insert (Ibidi) and left to adhere overnight in medium with 0.1% serum. The day after, medium was replaced with the CM collected from fibroblasts diluted 1:1 in fresh medium and left for 6 h before removing the inserts and starting the image acquisition using a Cell-IQ SLF instrument as already described [37].

### Animal studies

In vivo studies to characterize the immortalized fibroblasts were performed as already described [8, 30]. Experiments were approved by Ethics Committee for Animal Experimentation of the Fondazione IRCCS Istituto Nazionale dei Tumori (Milan, Italy) according to EU directive 2010/63/EU. CD1-Nude female mice, 5–10 weeks old, were divided in uniform groups on the basis of their weight. No engrafted animal was excluded from the analysis.

For tumorigenic assays, viable A549 tumor cells (5 × 10^5^) were exposed to CM of fibroblasts transfected with miR-C or miR-16 or CM supplemented with HGF-neutralizing antibody. After 2 days, cancer cells were injected subcutaneously (s.c.) into both flanks of nude mice with Matrigel 1:1 *v*/*v* (BD Biosciences) in a final volume of 200 μl. For co-injection experiments, A549 cells (1 × 10^3^) were injected s.c. with fibroblasts transfected with miR-C or miR-16. At the end of the observation period or when the tumor volume reached at least 300 mm^3^, lungs were removed and dissociated for the analysis of disseminated cancer cells as previously described [8].

## Results

### Identification of miRNAs regulating fibroblast pro-tumorigenic potential

**Fig. 1:**
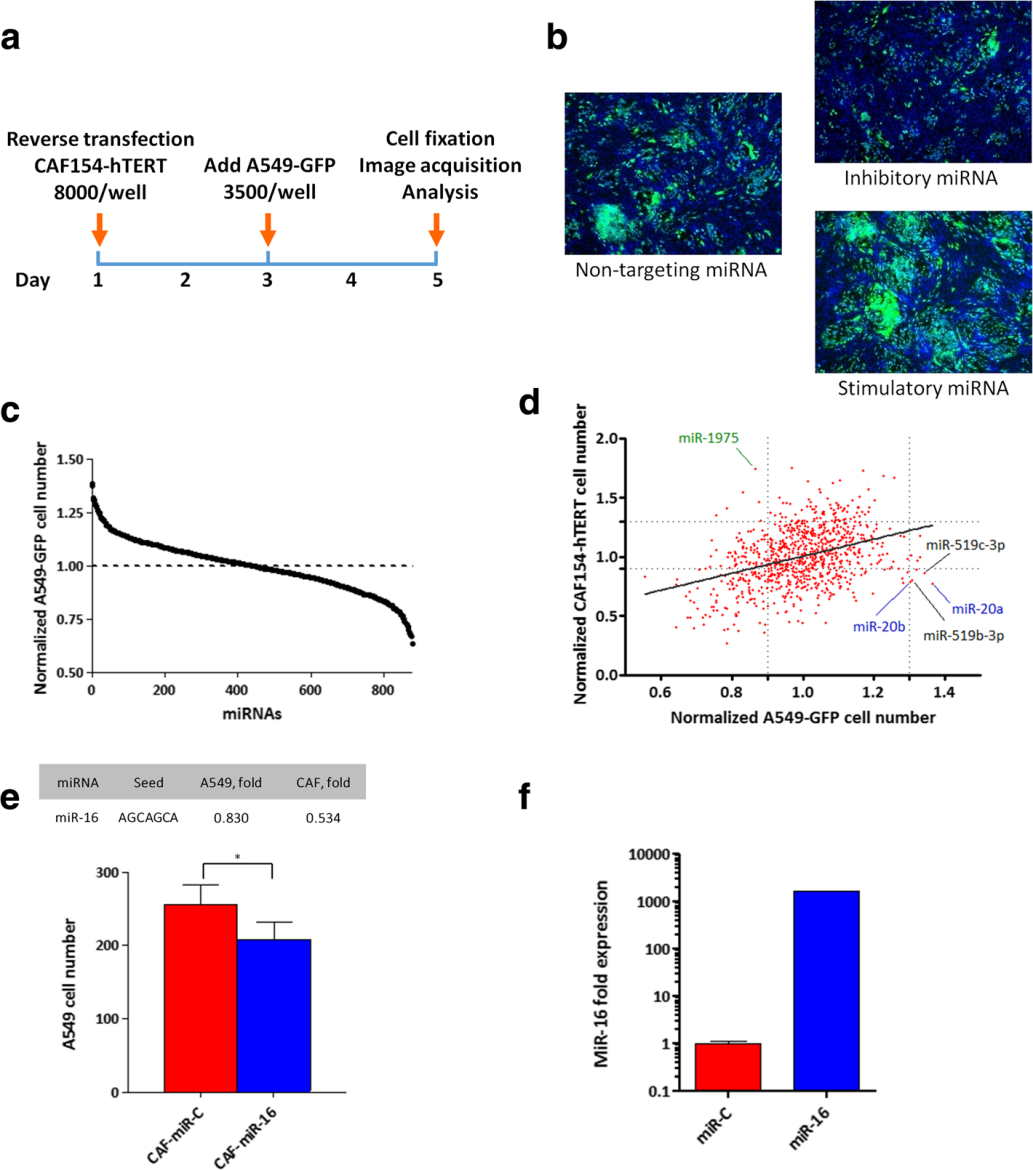
High-throughput screening to identify miRNAs that modulate the pro-tumorigenic potential of cancer-associated fibroblasts. **a** Schematic of the HTS: 8000 CAFs/well were seeded in 96-well plates and reverse transfected with a library of human miRNA mimics composed of 988 mature miRNAs (875 unique sequences). After 48 h, A549-GFP lung cancer cells were added (3500 cells/well) and further cultured for 48 h. Nuclei were then stained with Hoechst 33342, and automated fluorescence microscopy analysis was performed to quantify the total number of fibroblasts (GFP-negative) and A549 (GFP-positive) cells. Two independent screenings were performed; plates were normalized for the median of the samples of each plate and miRNAs were scored for their capacity to increase or decrease A549 cell growth. **b** Representative images of the screening showing cells transfected with a miRNA inhibiting the growth of the A549 cells (upper panel), a control miRNA (middle panel), and a miRNA stimulating the proliferation of A549 cells (lower panel). **c** Summary of the screening results, showing the distribution of A549 cell number after transfection with the miRNA library. **d** Effect of miRNAs on CAF154-hTERT fibroblasts and co-cultured A549 cell number (Spearman *r* = 0.35). Not all the miRNAs displayed the same effect on A549 and CAF154-hTERT cells. For example, miR-20a, miR-20b, miR-519b-3p, and miR-519c-3p inhibited the proliferation of CAF154-hTERT cells (< 0.9-fold) and simultaneously stimulated the proliferation of co-cultured A549 cells (> 1.3-fold). Contrarily, miR-1975 stimulated CAF154-hTERT fibroblast proliferation (> 1.3 fold), whilst inhibiting that of A549 cells (< 0.9-fold). **e** Effect of miR-16 on A549 and CAF154-hTERT cell proliferation in the HTS (upper panel; values normalized to controls) and validation of the effect of miR-16 transfection in CAF154-hTERT cells on co-cultured A549 cell growth (lower panel). In the latter case, four images per well were taken in different independent fields and GFP-positive cells automatically counted with the integrated Cell-IQ software. The averages of A549 cells from three wells were compared (*p* = 0.0235). **f** CAF154-hTERT fibroblasts were analyzed by real-time PCR 72 h after transfection with miR-C or miR-16 to evaluate miR-16 levels

To study the effect of individual miRNAs on fibroblast pro-tumorigenic features, we performed a high-throughput screening employing the patient-derived CAF154-hTERT cell line (see the “Methods” section for a detailed description). Fibroblasts were transfected with a library of 875 unique mature miRNAs, and after 48 h, GFP-expressing A549 lung cancer cells were added (Fig. 1a). MiRNAs were ranked on the basis of their capability to inhibit or stimulate the growth of co-cultured A549-GFP cells (Fig. 1b, c). Although the correlation was not very marked (Spearman *r* = 0.35), a significant number of miRNAs had the same effect on both A549 and CAF154-hTERT cell growth (Fig. 1d). However, a number of miRNAs produced opposite outcomes on the growth of these cell lines (Fig. 1d).

The 60 miRNA candidates displaying the strongest stimulatory effect on co-cultured A549 cells and the top 60 miRNAs which resulted in an inhibitory effect were then clustered according to their predicted ability to interact with the same mRNAs. Using this approach, these miRNAs could be grouped into six pro-stimulatory (Table 1) and five pro-inhibitory (Table 2) clusters, characterized by common seed regions.

**Table 1:** Clusters of miRNAs displaying a stimulatory effect on A549 cells in the HTS

Stimulatory miRNAs					
#1	#2	#3	#4	#5	#6
hsa-miR-302b (1.221)	hsa-miR-519d (1.307)	hsa-miR-519c-3p (1.305)	hsa-miR-199a-5p (1.214)	hsa-miR-146b-5p (1.261)	hsa-miR-520 h (1.191)
hsa-miR-302c (1.374)	hsa-miR-106a (1.238)	hsa-miR-519a (1.308)	hsa-miR-199b-5p (1.225)	hsa-miR-146a (1.266)	hsa-miR-520 g (1.257)
hsa-miR-302d (1.182)	hsa-miR-106b (1.288)	hsa-miR-519b-3p (1.291)			
hsa-miR-302a (1.250)	hsa-miR-20a (1.317)				
hsa-miR-520e (1.219)	hsa-miR-93 (1.384)				
hsa-miR-520b (1.215)	hsa-miR-20b (1.285)				
hsa-miR-520c-3p (1.274)	hsa-miR-17 (1.246)				
hsa-miR-372 (1.242)					
hsa-miR-520d-3p (1.278)					
hsa-miR-373 (1.188)					

Relative growth of A549 cells co-cultured with fibroblasts transfected with the indicated miRNAs is shown in brackets

**Table 2:** Clusters of miRNAs displaying an inhibitory effect on A549 cells in the HTS

Inhibitory miRNAs				
#1	#2	#3	#4	#5
hsa-miR-16 (0.729)	hsa-miR-28-5p (0.730)	hsa-miR-193a-3p (0.797)	hsa-miR-429 (0.689)	hsa-miR-620 (0.795)
hsa-miR-497 (0.783)	hsa-miR-708 (0.681)	hsa-miR-193b (0.743)	hsa-miR-200b (0.754)	hsa-miR-1270 (0.797)
hsa-miR-15b (0.787)			hsa-miR-200c (0.797)	

Relative growth of A549 cells co-cultured with fibroblasts transfected with the indicated miRNAs is shown in brackets

### Smoking correlates with reduced levels of miR-16 in lung fibroblasts

In an attempt to identify clinically relevant miRNAs, we focused on cluster #1 (Table 2) of the inhibitory miRNAs, which contains miR-16. In fact, by analyzing the levels of this miRNA in 47 primary cell lines of lung fibroblasts established from fresh tumor biopsies obtained from lung cancer patients (including 21 matched samples from both cancer tissue—cancer-associated fibroblasts (CAFs)—and non-involved lung parenchyma—normal fibroblasts (NFs)—for a total of 26 CAF lines and 21 NF lines), we explored the correlations between miR-16 expression levels and clinical parameters (Table 3). In detail, miR-16 levels in fibroblasts were not statistically associated with sex or age of patients, tissue of origin (cancer vs. normal), histology, and stage or grade of disease, but lower levels of miR-16 were detected in fibroblasts from smoke-exposed lungs compared to fibroblasts from non-exposed lungs [median intensity value 860 (IQ range 310–1437) vs. 1470 (IQ range 733–2120); *p* = 0.215], and in NF, this difference was statistically significant [324 (223–972) vs. 1589 (1494–2237); *p* = 0.006]. Interestingly, strongly reduced levels of miR-16 were also detected in fibroblasts from patients with chronic obstructive pulmonary disease (*p* = 0.048), in particular in NF (*p* = 0.004).

**Table 3:** Patients’ characteristics

Subgroup	No. of subjects	miR16 levels^a^					
		All	*p* value	CAF	*p* value	NF	*p* value
Sex							
Male	19	925 [441–2120]	0.746	925 [487–1961]	0.570	1180 [312–2109]	0.855
Female	7	1330 [966–1641]		1314 [1012–2427]		1347 [700–1494]	
Age							
< 70	15	1227 [384–1979]	0.567	1307 [649–2069]	0.750	1120 [252–1774]	0.470
> 70	11	1105 [528–2120]		919 [531–2095]		1519 [606–1987]	
Histology							
Adenocarcinoma	19	1112 [475–1519]	0.488	1105 [733–1466]	0.960	1120 [290–1519]	0.147
Others	7	2078 [493–2475]		1307 [487–2966]		2216 [916–2442]	
Stage							
I–II	9	1105 [528–1520]	0.566	800 [486–1224]	0.520	1433 [762–1537]	0.950
III–IV	17	1227 [384–2295]		1307 [733–2456]		1120 [281–2120]	
Grade							
G2	4	1217 [916–1479]]	0.946	1390 [1261–2230]	0.628	734 [277–1219]	0.490
G3	16	922 [452–1658]		922 [553–2003]		1094 [286–1572]	
Smoking							
Current	12	860 [310–1437]	0.215	1074 [722–2584]	0.980	324 [223–972]	0.006
Former^b^/Never	14	1470 [733–2120]		1126 [517–1590]		1589 [1494–2237]	
COPD (GOLD)							
0	15	1553 [826–2467]	0.048	1307 [616–2711]	0.456	2078 [1494–2412]	0.004
1–2	10	800 [324–1233]		860 [508–1331]		349 [281–1120]	

^a^Data are median [IQR] of gTotalGeneSignal (Agilent Array)

^b^Former smoker > 12 months

We therefore speculated that inflammation and smoke-related miR-16 reduction found in patients could contribute to generation of a microenvironment conductive to cancer proliferation and aggressiveness, while its restoration in fibroblasts could reduce the growth of cancer cell. Accordingly, transfection of CAF154-hTERT cells with miR-16 resulted in the inhibition of both fibroblast and adjacent A549 cell proliferation (Fig. 1e, upper panel) in our HTS, and the effect on A549 cells co-cultured with transfected fibroblasts was further confirmed in independent experiments (Fig. 1e (lower panel), f). Importantly, also, miR-497 and miR-15b, which target the same seed region of miR-16, were found among the top candidates (cluster #1, Table 2), and the other miRNAs belonging to the same family (miR-195, miR-15a, and miR-424), even if less efficiently, all showed an inhibitory effect on A549 cells (0.902, 0.837, and 0.834 normalized A549 cell number, respectively).

### Expression of miR-16 affects CAF secretome and massively decreases HGF levels

To investigate the mechanisms underlying the inhibitory effect of miR-16 on cancer cells, we first considered the possibility that miR-16 could be released from fibroblasts and taken up by A549 cells. However, with experiments on transfected fibroblasts conditioned medium (CM), we estimated a concentration of released miR-16 ranging between 0.1 and 0.05 nM and found that similar amounts of miRNA did not influence A549 cell proliferation (data not shown).

**Fig. 2:**
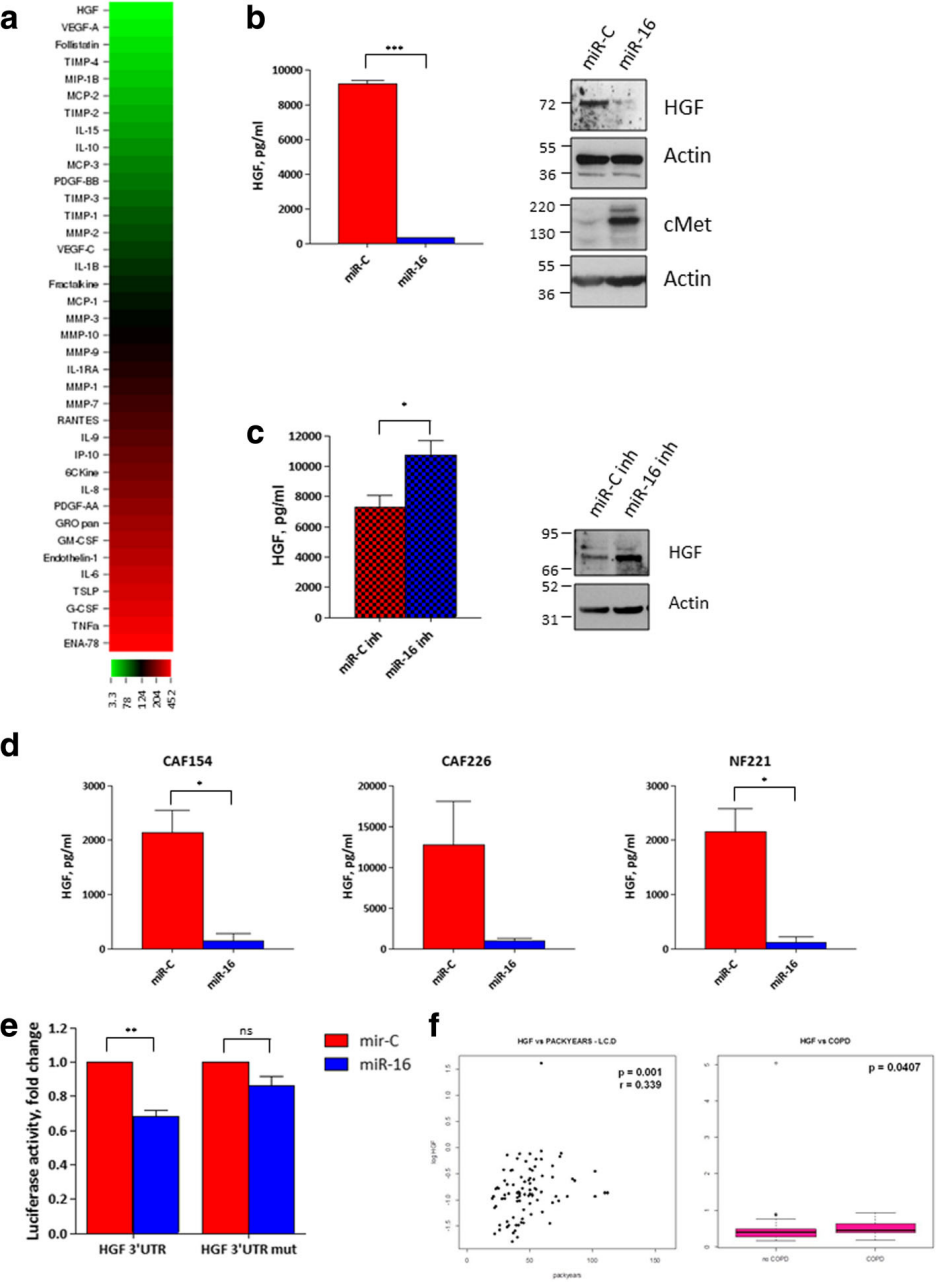
Ectopic expression of miR-16 affects the CAF secretome and reduces HGF levels. **a** CAF154-hTERT fibroblasts (1.5 × 10^5^ cells/well) were reverse transfected with miR-16 in 12-well plates, and medium was harvested 72 h later, clarified by centrifugation, and analyzed with a fluorescence-based multiplex assay. Overall, 91 unique factors were quantified in the conditioned medium. The concentration of each soluble factor after miR-16 transfection is expressed as a percentage compared of miR-C-transfected CM. **b** Concentration of HGF in CM collected 72 h after transfection of 1.5 × 10^5^ CAF154-hTERT fibroblasts with miR-C and miR-16 (*n* = 3; *p* < 0.0001). Western blot showing the levels of HGF and cMet in CAF154-hTERT fibroblasts 72 h after transfection with miR-16 and non-targeting miR-C. Actin is shown as a loading control. **c** HGF levels in the CM and western blot of CAF154-hTERT fibroblasts transfected as in **c** with a control miRNA (miR-C inh) and miR-16 inhibitor (miR-16 inh; *n* = 3, *p* = 0.0358). **d** The effect of miR-16 ectopic expression was determined in CM collected from a number of primary patient-derived fibroblasts (CAF154, *n* = 3, *p* = 0.0259; CAF226, *n* = 2; NF221, *n* = 3, *p* = 0.0316). HGF concentration was evaluated by ELISA. **e** The direct targeting of miR-16 on the 3′UTR of the HGF mRNA was determined by luciferase assay performed by transfection of 293 T cells with 100 nM miRNAs together with the pMirTarget HGF 3′UTR and a *Renilla*-expressing plasmid. The luciferase expression was evaluated 24 h after transfection by dual-luciferase assay and normalized to *Renilla* expression. To test the specificity of miR-mRNA interaction, the assay was performed also with a mutated version of the pMirTarget HGF 3′UTR in which the putative binding site of miR-16 was mutagenized (Additional file 3: Figure S3; **p = 0.002; ns, non-significant). **f** Correlation between circulating HGF concentration and smoke exposure (pack-years), *r* = Spearman correlation *p* value (left), and circulating HGF concentration and COPD, *p* = Wilcoxon test *p* value (right) in healthy heavy smokers (*n* = 90)

We next investigated therefore whether miR-16 ectopic expression in CAF154-hTERT fibroblasts could affect the secretome profile of the transfected cells, thereby influencing the growth of A549 cells. CAF154-hTERT fibroblasts were transfected with control miRNA (miR-C) or miR-16, and CM analyzed after 72 h by multiplex array to quantify 91 unique soluble factors, belonging to three human protein sets: (i) cytokines, (ii) MMPs and TIMPs, and (iii) angiogenetic factors. We observed marked differences in the CM obtained from fibroblasts transfected with miR-16, finding 26 out of 91 soluble factors with concentrations < 75 or > 125% compared to miR-C transfected fibroblasts (Fig. 2a).

Among the perturbed factors, HGF was the most affected by miR-16 transfection and was almost completely depleted in the CM (Fig. 2a, b). Moreover, HGF expression was reduced also at the cellular level (Fig. 2b, right), suggesting a reduction of its expression rather than an inhibition of its release. Conversely, the levels of its cognate receptor cMet increased, compatibly with a stalled turnover due to reduced paracrine or autocrine stimulation (Fig. 2b, right). Importantly, inhibition of miR-16 resulted in an opposite effect and caused the accumulation of HGF both in the CM (Fig. 2c, left) and intracellularly (Fig. 2c, right). To rule out the possibility that miR-16 regulates HGF levels in a cell-type-specific manner, we transfected a number of patient-derived primary fibroblasts with miR-16 and consistently found a striking reduction of HGF levels in the CM (Fig. 2d), while miR-16 inhibition reproducibly increased HGF levels (Additional file 4: Figure S4). We checked whether miR-16 directly targets the HGF 3′UTR by luciferase assay and found a partial, yet significant, reduction of the luciferase activity when cells were transfected with miR-16 (Fig. 2e). Importantly, when the sequence corresponding to the seed region of miR-16 was mutated in the HGF 3′UTR (Additional file 3: Figure S3), the effect of the miRNA was almost completely abrogated (Fig. 2e), indicating a direct targeting of miR-16 on HGF mRNA.

Finally, we reasoned that the microenvironmental changes related to miR-16 reduction, which we previously observed in lung-derived fibroblasts (Table 3), could also potentially influence systemic levels of HGF. Interestingly, analysis of plasma samples from healthy heavy smokers enrolled in a CT-screening program for early lung cancer detection revealed a strong correlation between smoke exposure or COPD and circulating levels of HGF (*p* = 0.001 and *p* = 0.041 respectively, Fig. 2f). No correlation was found with age (data not shown).

### MiR-16 expression indirectly decreases FGFR-1 levels and inhibits its signaling pathway further contributing to HGF reduction

**Fig. 3:**
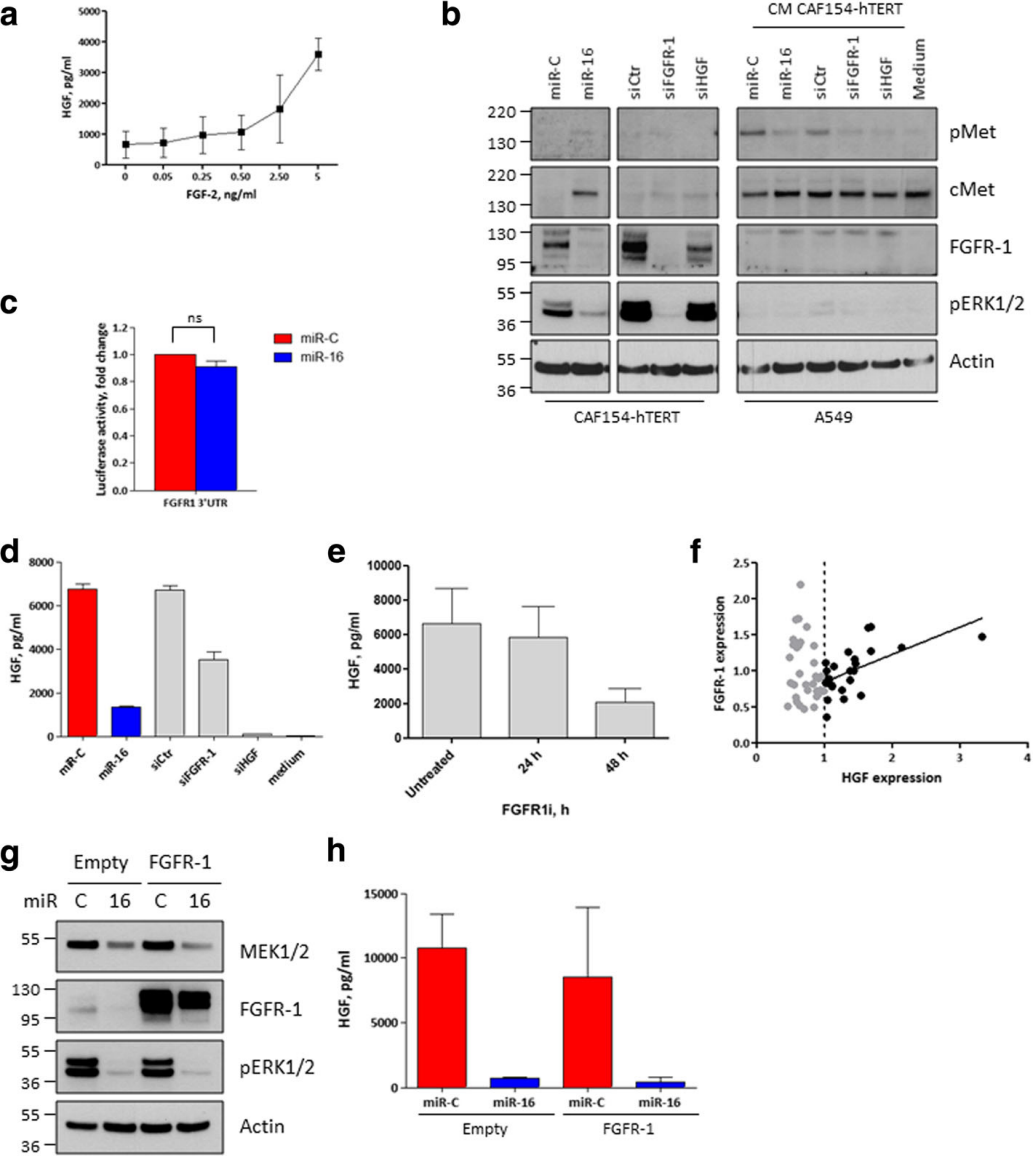
The FGFR-1 receptor regulates HGF secretion, and it is targeted by miR-16. **a** CAF154-hTERT fibroblasts (3 × 10^5^ cells/well) were serum-starved for 48 h and then stimulated with increasing doses of FGF-2. CM was collected after 24 h and analyzed by ELISA to evaluate the levels of secreted HGF. **b** CAF154-hTERT fibroblasts were transfected with the non-targeting miR-C, miR-16, a control siRNA, or a siRNA targeting FGFR-1 or HGF. After 72 h, the CM was collected and, together with a non-conditioned medium, used to stimulate the A549 cells. Western blot was performed on transfected CAF154-hTERT fibroblasts and on stimulated A549 cells to detect the activated state of cMet and its total levels, the levels of FGFR-1, and the activated form of ERK1/2. Actin is shown as a loading control. **c** Luciferase assay performed as in Fig. 2e, by using 293 T cells transfected with the pMirTarget FGFR-1 3′UTR and a *Renilla*-expressing plasmid. The FGFR-1 3′UTR displays two putative miR-16 binding sites (Additional file 3: Figure S3). **d** Levels of HGF in CM of CAF154-hTERT fibroblasts transfected with the control miR-C and miR-16 or a control siRNA and siRNA specific for FGFR-1 or HGF. Non-conditioned medium is shown as a negative control. **e** CAF154-hTERT fibroblasts were treated with 10 μM FGFR-1 inhibitor SU5402 and CM collected at the indicated times to evaluate HGF concentration by ELISA. The graph is representative of two independent experiments. **f** Correlation between the expression of HGF and FGFR-1 in primary fibroblasts derived from lung cancer patients and expressing high levels of HGF (*R*^2^ = 0.3607, slope = 0.3797 ± 0.1054). **g** CAF154-hTERT fibroblasts were transduced with lentiviral particles to stably express FGFR-1 and transfected with control miR-C and miR-16. Western blot was performed 72 h after transfection to detect MEK1/2 and FGFR-1 levels and the activated form of ERK1/2. Actin is shown as a loading control. **h** ELISA performed to evaluate the levels of HGF in the CM of cells transfected as described in **g**

Taking into account the strong effect of miR-16 on HGF secretion (Fig. 2a, b) and the only partial reduction of the luciferase activity in the HGF 3′UTR reporter assay (Fig. 2e), we also considered additional inhibitory mechanisms and focused on the FGFR-1 receptor. Stimulation of this receptor by means of its cognate ligand FGF-2 resulted in the accumulation of HGF in the CM (Fig. 3a) while miR-16 transfection strongly reduced the levels of FGFR-1 (Fig. 3b), but not of its cognate ligands FGF-1 and FGF-2 (Additional file 5: Figure S5), and it hindered the activation of the FGFR-1 downstream mediators ERK1/2 (Fig. 3b). Of note, miR-16-induced reduction of FGFR-1 apparently did not stem from a direct effect of this miRNA on FGFR-1 mRNA, as judged by the lack of effect of miR-16 on FGFR-1 3′UTR in a luciferase reporter assay (Fig. 3c). Nonetheless, the transfection of CAF154-hTERT cells with miR-16 or an FGFR-1-specific siRNA both resulted in HGF reduction (Fig. 3d). Moreover, treatment with a specific inhibitor of FGFR-1 caused a time-dependent reduction of HGF in the CM (Fig. 3e).

The stimulation of A549 cells with CM collected from control CAF154-hTERT cells caused a clear and rapid activation of the cMet receptor (Fig. 3b), while CM collected from the same cells transfected with miR-16 and with siRNAs directed against FGFR-1 and HGF failed to trigger the phosphorylation of cMet (Fig. 3b). Importantly, we also observed a direct correlation in patient-derived fibroblasts between HGF and FGFR-1 expression, which was however evident only in HGF high-expressing fibroblasts (HGF > 1, Fig. 3f).

We then asked whether the restoration of FGFR-1 levels was sufficient to prevent the effect of miR-16 on HGF and therefore transduced CAF154-hTERT cells to ectopically express FGFR-1 (Fig. 3g). Despite high levels of FGFR-1, miR-16 still markedly reduced the secretion of HGF (Fig. 3h), suggesting the presence of other miR-16 targets which control HGF expression downstream FGFR-1. Interestingly, miR-16 transfection abrogated ERK1/2 activation also in the presence of FGFR-1 over-expression and this correlated with the downregulation of the MEK1/2 kinases (Fig. 3g), which are responsible for ERK1/2 phosphorylation and could therefore represent additional targets of miR-16.

### MEK1 is a direct target of miR-16 and regulates HGF levels in fibroblast CM

**Fig. 4:**
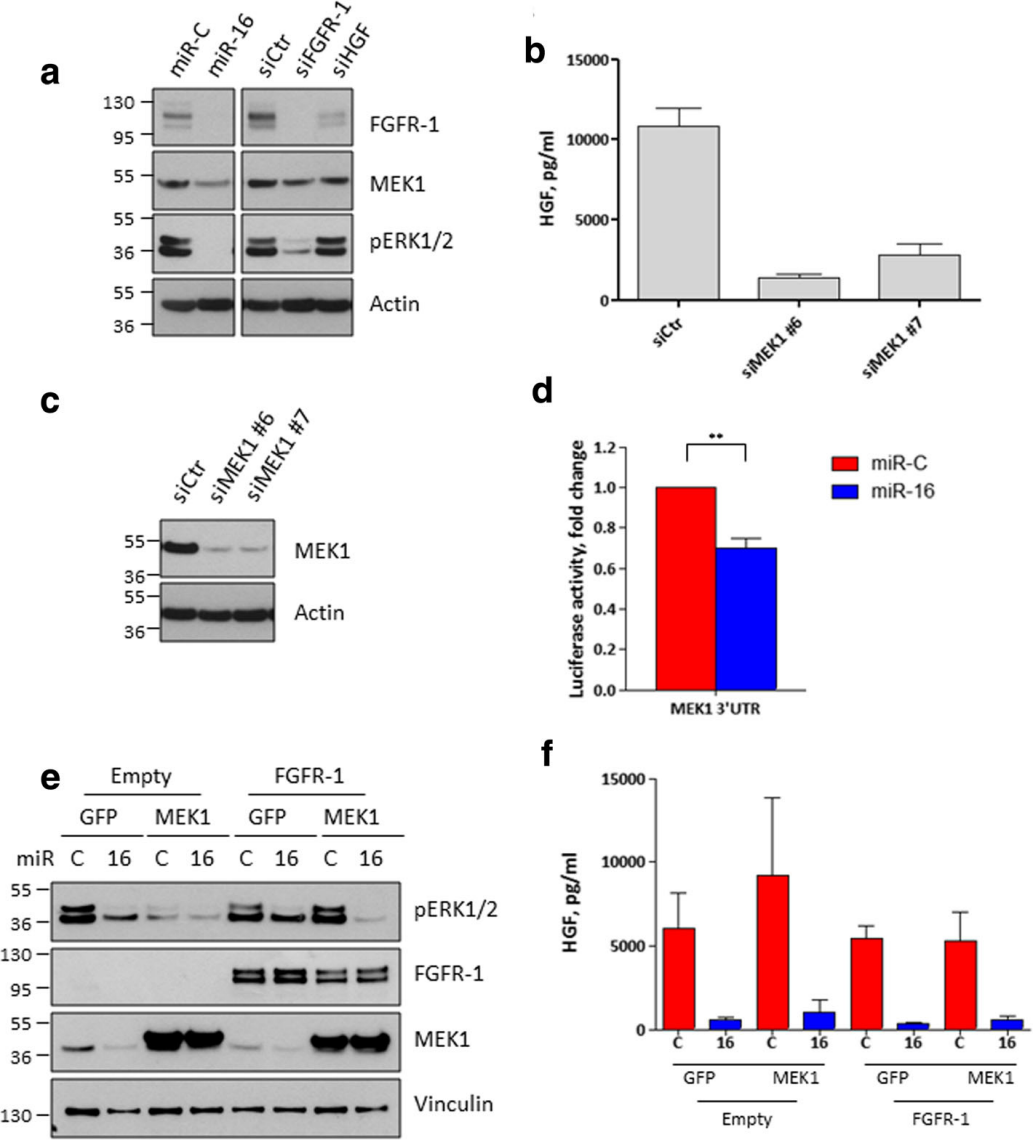
MEK1 regulates HGF secretion and is regulated by miR-16. **a** CAF154-hTERT cells were transfected with miR-16 and control miR-C, a non-targeting siRNA, and siRNAs targeting FGFR-1 or HGF. After 72 h, fibroblasts were collected and analyzed by western blot to detect the total levels of FGFR-1 and MEK1 and the activated form of ERK1/2. Actin is shown as a loading control. **b** The CAF154-hTERT fibroblasts were transfected with two different siRNAs targeting MEK1; after 72 h, the CM was collected to quantify the levels of secreted HGF and **c** cells analyzed by western blot. **d** Luciferase assay performed as described in Fig. 2e to test direct targeting of MEK1 3′UTR by miR-16 (**p = 0.0084). The miR-16 binding site in the MEK1 3′UTR is shown (Additional file 3: Figure S3). **e**, **f** Cells transduced as described in Fig. 3g were further transduced with lentiviral particles to ectopically express MEK1 or GFP, as a control, and transfected with control miR-C and miR-16. The experiment was stopped after 72 h to detect by western blot the cell levels of FGFR-1, MEK1, activated ERK1/2, and actin, as a loading control (**e**), and to evaluate, by ELISA, the concentration of HGF in the CM (**f**)

To confirm the involvement of MEK1 in the miR-16-dependent regulation of HGF, we transfected CAF154-hTERT cells with this miRNA and found that MEK1 was indeed reduced (Fig. 4a). Moreover, silencing of MEK1 (but not MEK2) prevented HGF accumulation in the CM (Fig. 4b, c and data not shown). By using a luciferase 3′UTR reporter assay, we confirmed that MEK1 is a direct target of miR-16 (Fig. 4d) and it represents a determinant of HGF secretion in the CM (Fig. 4b). Similar to what was observed for FGFR-1, ectopic expression of MEK1 is not sufficient to prevent the miR-16-dependent downregulation of HGF (Fig. 4e, f). Altogether, our data show that miR-16 acts as potent inhibitor of HGF by targeting directly and indirectly several mediators of the FGFR-1 pathway which in turn controls HGF expression.

### Fibroblast miR-16 levels regulate motility features of adjacent cancer cells

**Fig. 5:**
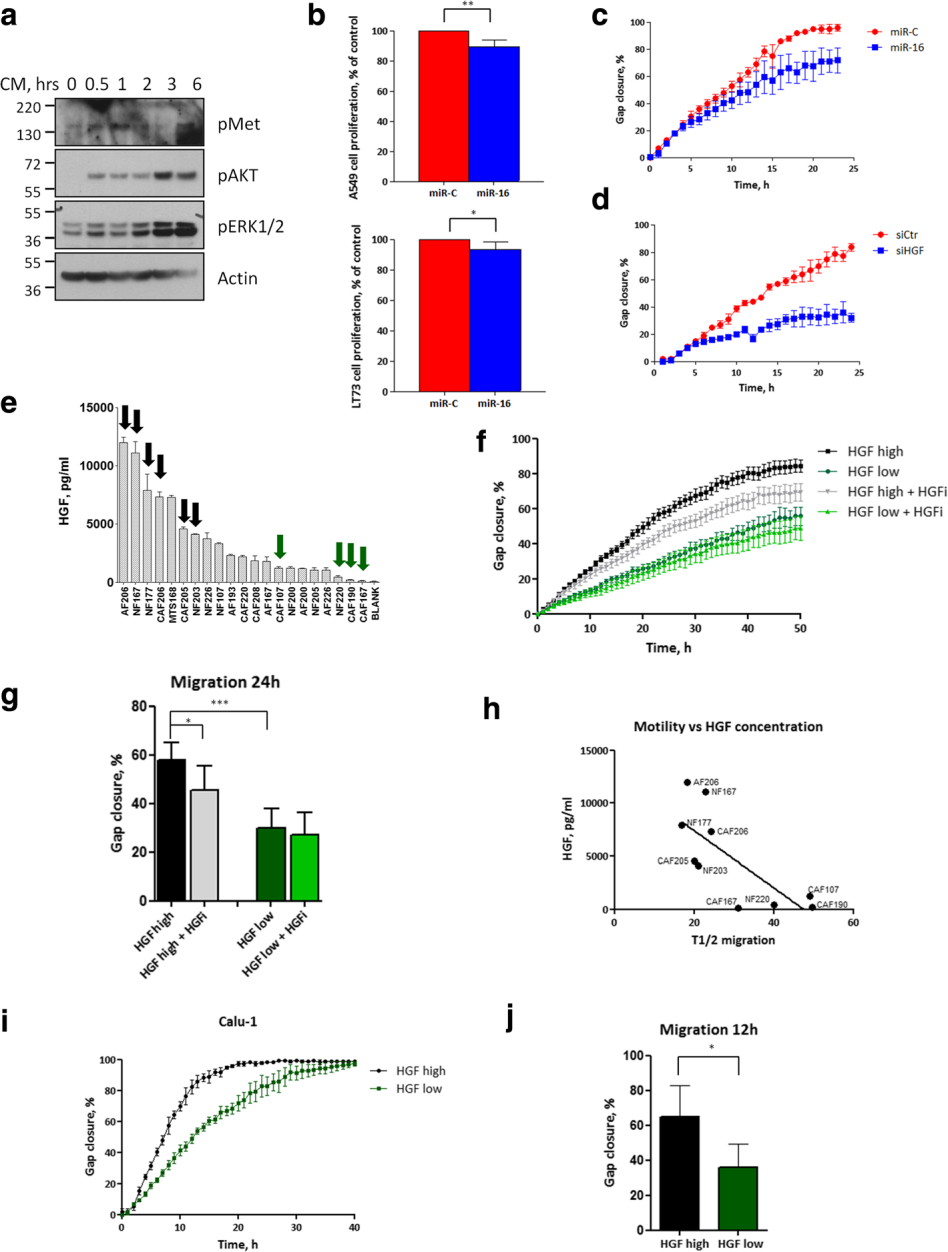
The CM derived from miR-16-transfected fibroblasts displays reduced pro-tumorigenic properties. **a** Serum-starved A549 cells were stimulated for the indicated periods with the CM collected from CAF154-hTERT fibroblasts and diluted 1:2 in medium without serum. Western blot was performed to detect the activation of cMet, AKT, and ERK pathways. Actin is shown as a loading control. **b** A549 (upper panel) and LT73 (bottom panel) cells were stimulated with CM derived from CAF154-hTERT fibroblasts transfected with control miR-C or miR-16, and cell proliferation was measured 72 h later by CTG (**p = 0.0066, *n* = 5; *p = 0.0386). **c** A549 cells stimulated as in **b** were employed in wound-healing experiments. **d** A549 cells were stimulated with CM collected from CAF154-hTERT fibroblasts transfected with a siRNA specific for HGF. **e** HGF concentration in the CM of a panel of primary patient-derived fibroblasts. Arrows indicate CM media used in **f** and **i** (black arrows for high-concentration and green arrows for low-concentration of HGF). **f** Wound-healing experiments performed as in **c** with A549 cells stimulated with CM shown in **e**, with or without an HGF-neutralizing antibody (HGFi). **g** Results of migration experiments (**f**) after 24 h of migration (*p = 0.0355; ***p = 0.0004). **h** Time necessary to close half of the gap (T1/2) was plotted together with the concentration of HGF in the CM (*R*^2^ = 0.5647, slope = − 269.0 ± 83.51). **i** Migration experiments were performed with Calu-1 cells stimulated with a fibroblast-derived CM containing high levels of HGF (CAF206) and one with low levels of the cytokine (CAF190). **j** Results of migration experiments (**i**) after 12 h of migration in three independent experiments (*p = 0.0241)

The stimulation of A549 cells with CM collected from CAF154-hTERT cells had a pleiotropic effect, activating different pathways known to enhance survival and aggressiveness of cancer cells. In fact, in time-course experiments, we observed a rapid activation of cMet, AKT, and ERK1/2 (Figs. 5a and 3b). Therefore, we investigated whether the stimulation with CM could promote proliferation and motility of A549 cancer cells in a miR-16-dependent manner. CM collected from CAF154-hTERT cells transfected with miR-16 displayed a reduced capacity to promote proliferation of A549 cancer cells (Fig. 5b, upper panel) and also induced a small, but significant, difference in proliferation in another cell line previously shown to be responsive to microenvironment cues [30] (LT73, Fig. 5b, bottom panel). Additionally, A549 cells stimulated with the same CM showed reduced migratory capacity in wound-healing experiments (Fig. 5c). Of note, the pro-migratory property of HGF was assessed in our settings also by using CM collected from CAF154-hTERT cells transfected with a siRNA targeting HGF. This caused a marked delay of A549 cell migration compared to controls (Fig. 5d), confirming a central role for HGF in this setting. A panel of CM collected from primary patient-derived fibroblasts was then characterized for the concentration of HGF (Fig. 5e). Conditioned media collected from fibroblasts containing low (green arrows) and high (black arrows) levels of HGF (Fig. 5e) were employed to stimulate A549 cells in wound-healing experiments in the presence or absence of an HGF-neutralizing antibody (Fig. 5f). A549 cells stimulated with CM containing high levels of HGF migrated significantly more rapidly compared to those stimulated with low-HGF CM (Fig. 5f, g), and the HGF-neutralizing antibody partially abrogated this effect (Fig. 5f, g), thus confirming the role of fibroblast-derived HGF in promoting motility of cancer cells. Moreover, the migration speed (expressed as time point at which the gap is reduced to half of the original area, T1/2) was inversely correlated to the concentration of HGF in the CM (Fig. 5h). To verify whether the HGF-dependent pro-migratory effect of fibroblast CM was specific for A549 cells or generally applicable to other cancer cell lines, we repeated these experiments with squamous lung cancer Calu-1 cells. Migration experiments confirmed that cell motility depends on the HGF concentration present in CM (Fig. 5i–j).

### Over-expression of miR-16 in fibroblasts and inhibition of soluble HGF hinder cancer aggressiveness in vivo

**Fig. 6:**
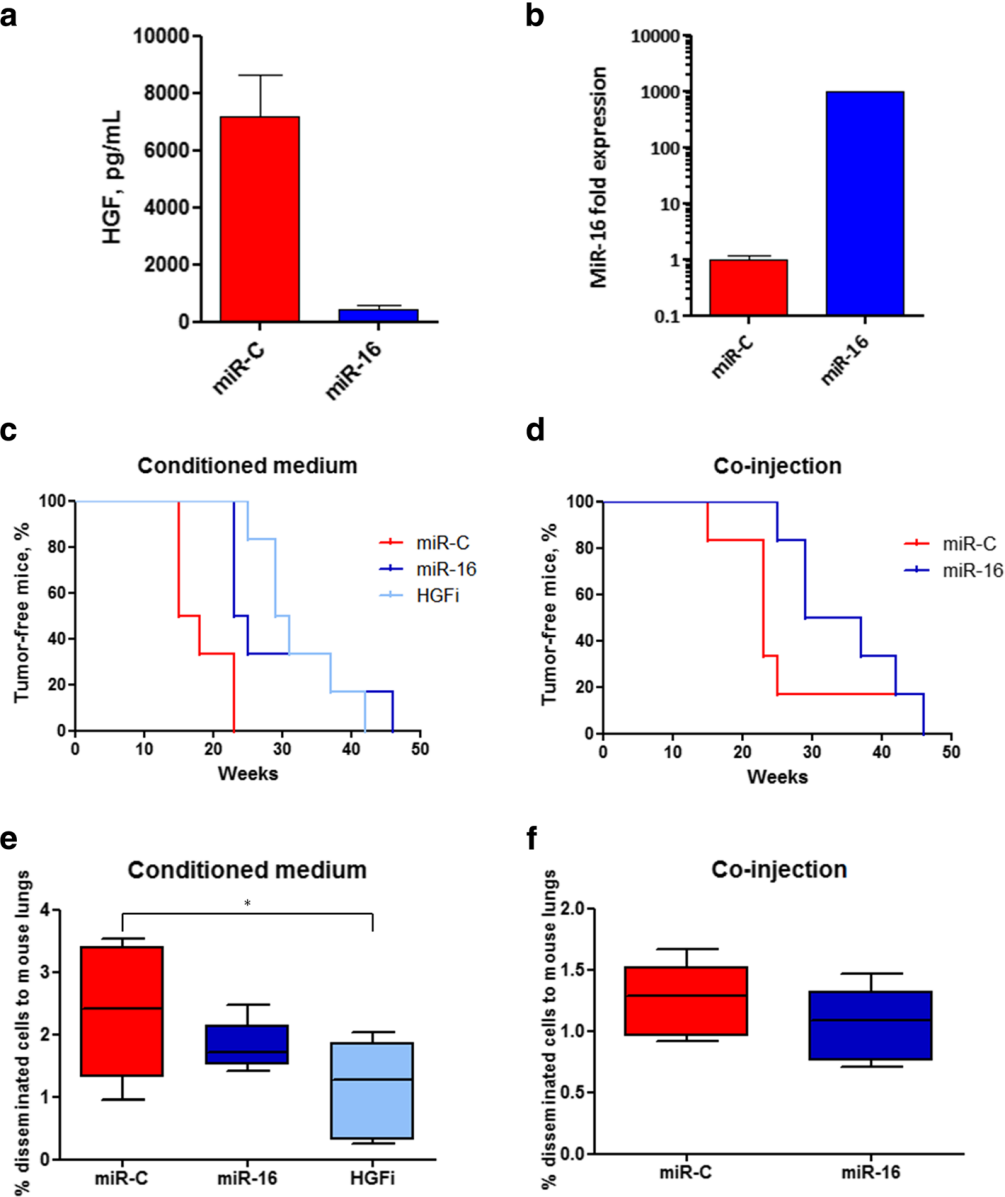
MiR-16 affects the pro-tumorigenic properties of the fibroblasts in vivo. **a** HGF levels in the CM medium employed and **b** miR-16 expression in the CAF154-hTERT fibroblasts were evaluated by ELISA and real-time PCR, respectively. **c** A549 cells (5 × 10^5^ cells) were injected in the flanks of immunosuppressed nude mice after 24 h culturing in CM (1:2) collected from CAF154-hTERT fibroblasts transfected with mir-C, with or without a neutralizing anti HGF antibody (HGFi), and miR-16 (*n* = 6). Mice were considered engrafted when tumor volume reached 100 mm^3^ (Gehan-Breslow-Wilcoxon test: miR-C vs miR-16 *p* = 0.0090, miR-C vs HGFi *p* = 0.0014). **d** A549 cancer cells were subcutaneously injected in nude mice together with the fibroblasts described in **c** (ratio cancer cells/fibroblasts 1:3; Gehan-Breslow-Wilcoxon test: *p* = 0.0437). **e**, **f** Lungs collected from mice described in **c** and **b**, respectively, were collected and analyzed by FACS for the presence of metastatic human cells (**e** miR-C vs miR-16, *p* = 0.2997; miR-C vs HGFi *p = 0.0312; **f** miR-C vs miR-16, *p* = 0.0735)

Having shown that fibroblast miR-16 levels slightly affect the proliferation capacity of adjacent cancer cells and can strongly influence their migration, we investigated the relevance of the miR-16/HGF axis in vivo. Two experiments were performed by culturing the A549 cells with the CM collected from CAF154-hTERT fibroblasts transfected with miR-16 and control miR-C (Fig. 6a, b) before mouse engrafting (Fig. 6c) or by directly co-injecting the A549 cells with the transfected fibroblasts in nude mice (Fig. 6d).

In both cases, transfection with miR-16 of CAF154-hTERT fibroblasts delayed the ability of the A549 cancer cells to form nodules. Moreover, inhibition of HGF in CM by means of a neutralizing antibody showed an effect comparable to miR-16 transfection (Fig. 6c). Importantly, mouse lung analysis revealed that HGF inhibition also reduced the capacity of A549 cells to metastasize to lungs (Fig. 6e) in a significant manner while ectopic expression of miR-16 in CAF154-hTERT fibroblasts reduced the metastasizing potential of cancer cells, albeit not significantly (Fig. 6e, f).

## Discussion

Tumor microenvironment (TME) is increasingly perceived as a major determinant of cancer progression and aggressiveness [38]. Among the cellular components of the TME, stromal fibroblasts play a crucial role during tumorigenesis [39]. Here, we show that miR-16 levels in lung fibroblasts control HGF production in an FGFR1-dependent manner unraveling a novel mechanism of communication between stromal and neoplastic cells in lung cancer, with potential clinical implications.

Initially, we exploited a high-throughput screening strategy to evaluate the function of 875 unique mature miRNAs in regulating the pro-tumorigenic properties of lung fibroblasts. For the screening, we implemented a robust technical setup based on immortalized patient-derived cancer-associated fibroblasts (CAFs) and a lung cancer cell line previously shown to be responsive to microenvironment signals [30]. The choice of patient-derived CAFs, which are already endowed with intrinsic tumor promoting activity, was based on the hypothesis that this could lead to the identification of miRNAs that could either increase or decrease this potential. Accordingly, the screening identified several miRNAs that could be clustered by their predicted targets to identify potential master regulators of the cross-talk between stromal and cancer cells. Of note, the majority of miRNAs showed a similar effect on the growth of both fibroblasts and A549 cells, but still some displayed opposite effects. The latter observation, on the one hand, supports the idea that the small concentrations of miRNAs released from CAF154-hTERT fibroblasts were not responsible for the inhibitory/stimulatory A549 cell growth effect and, on the other, confirms previous findings demonstrating that the same miRNA can display opposite effects between CAFs and adjacent cancer cells through the modulation of fibroblast-derived soluble factors [40].

Among the identified candidates, we focused on miR-16, one of the prominent negative regulators of the pro-tumorigenic activity of fibroblasts in our assay and previously shown to have direct oncosuppressive properties in tumor cells [15, 16, 41] and to mediate tumor-stroma interactions in prostate cancer [17]. Importantly, the effects observed in our experiments could not be attributed to a direct effect of miR-16 on cancer cells, but rather to a modulation of fibroblast secretome. Several factors produced by CAFs are known to regulate cancer cell behavior through different mechanisms [42]. In particular in lung cancer, it has been suggested that CAF-produced IL-6 regulates chemoresistance [43] and stromal-derived TGFβ and IGF-II have both been shown to induce EMT and regulate stemness properties of cancer cells [30, 44], but little is known about the mechanisms underlying the modification of fibroblast secretome. We identified HGF as one of the most affected factors, strongly depleted in the CM of cells upon miR-16 transfection.

**Fig. 7:**
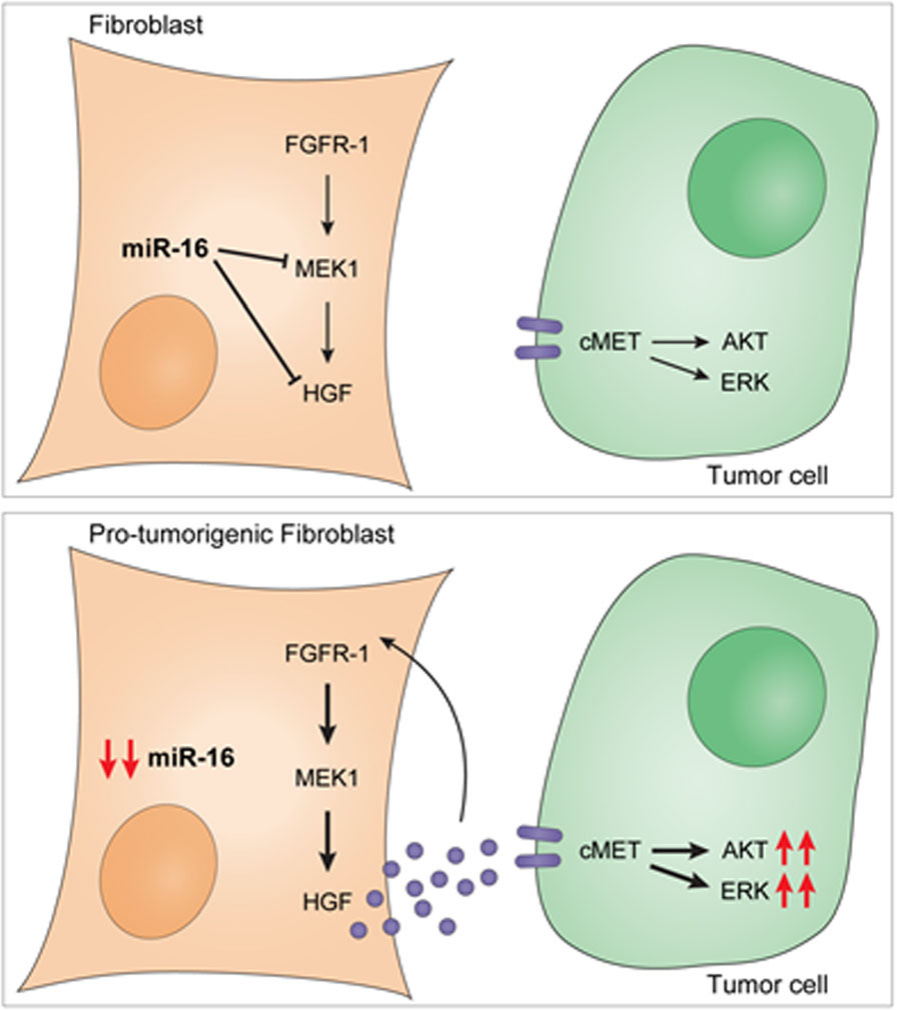
MiR-16 affects the pro-tumorigenic properties of the fibroblasts by controlling the levels of HGF in an FGFR-1- and MEK1-dependent fashion. According to our data, miR-16 reduces fibroblast HGF secretion by direct targeting of HGF itself and by inhibiting the FGFR-1 pathway, which in turn promotes HGF expression. In fact, even if not directly targeting the FGFR-1 mRNA, miR-16 reduces FGFR-1 protein levels and directly targets MEK1, which is crucial for FGFR-1 downstream signaling. Moreover, HGF seems to contribute to FGFR-1 expression. When miR-16 is reduced or lost, HGF is secreted by fibroblasts and contributes to cancer cell aggressiveness through the stimulation of cMet pathway. This activation results in increased proliferation and motility of the cancer cells. Interestingly, the presence of HGF seems to favor the increased levels of FGFR-1 supporting the idea that a cross-talk exists between cMet and FGFR-1 receptors

HGF has pleiotropic activities both in normal and in cancer cells, and its role in cancer has been widely documented [45]. The precise mechanism of regulation of its secretion within the lung cancer microenvironment and its relevance in lung cancer aggressiveness are still unknown. We demonstrated a direct targeting of the HGF transcript by miR-16, but this could not fully explain the extent of HGF reduction. In fact, miR-16 upregulation reduced FGFR-1 levels, and this further decreased HGF levels. Conversely, stimulation of FGFR-1 with its ligand FGF-2 resulted in HGF accumulation. Together with FGFR-1, other downstream mediators of its signaling pathway, including MEK1, were also identified as targets of miR-16 (Fig. 7). This suggested profound implications considering the potential paracrine effect of HGF on surrounding cancer cells in the TME.

To test the relevance of HGF production by fibroblasts in modulating cancer cell phenotype, we performed migration assays with different lung cancer cells testing the stimulatory potential of medium collected from several primary cell lines of lung fibroblasts isolated from lung cancer patients. A clear correlation between production of HGF and stimulation of cancer cell migration was observed. Furthermore, transfection with miR-16 abrogated the pro-migratory effect of cells with elevated HGF production strengthening even further the link between miR-16 and HGF in lung fibroblasts and highlighting the potential role of this mechanism in regulating aggressiveness of this deadly disease. This notion was confirmed in vivo where miR-16 expression in fibroblasts reduced their ability to induce cancer cells to form subcutaneous tumors and disseminate to lungs.

In our assays, we used fibroblast lines isolated both from cancerous or normal tissues (CAFs or NFs) and did not observe a different behavior depending on their origin, but rather on the levels of HGF produced. This is in accordance with previous observations reporting that in lung cancer patients, also “normal” fibroblasts can display an activated phenotype and possibly reflecting their origin from an organ heavily exposed to carcinogens in the setting of heavy smokers [12]. Curiously, in this respect, maternal-smoke-associated reduction of miR-16 in the placenta has been described [46]. Even if correlation between smoke exposure and miR-16 levels in clinical samples is at present interesting, future work should address the mechanistic basis of this correlation. Nonetheless, in our case series, we found a correlation between smoke exposure (and COPD) and reduced expression of miR-16 in lung fibroblasts derived from normal tissue raising the intriguing hypothesis that smoke-induced stromal modifications could pave the way for cancer development by stimulating the growth of incipient lesions. The lack of correlation in CAFs could be explained by the fact that when tumor lesions are established, the intense cross-talk between cancer cells and fibroblasts results in higher phenotypic heterogeneity among fibroblasts (and hijacking of different metabolic pathways) which may therefore not reflect anymore the original “smoke-associated” (or inflammatory) signature. An alternative hypothesis relates to the potential origin of CAFs from circulating precursors (rather than from “resident fibroblasts”; [47, 48]). In this scenario, a “smoking” signature in CAFs deriving from circulating precursors would in fact not necessarily be expected. Finally, systemic HGF levels resulted to be elevated by exposure to smoke in individuals at high-risk for lung cancer, therefore strengthening the existence of a novel additional mechanism by which smoking could promote cancer progression. Of note, downregulation of miR-16 levels in the circulation is also a feature of our recently validated circulating miRNA-based signatures with predictive and prognostic value in heavy smokers undergoing spiral CT screening for lung cancer [35, 49].

## Conclusions

In conclusion, we show here that miR-16 is a crucial mediator of HGF production by lung fibroblasts through regulation of different downstream targets including FGFR-1 and MEK1. Since production of HGF by lung fibroblasts regulates lung cancer cell aggressiveness, these findings have potential clinical implications. Taken together, our findings could imply the existence of a co-stimulatory loop whereby FGF-2 produced by cancer cells stimulates secretion of HGF by CAFs which in turn promotes migration and aggressiveness of cancer cells.

## Additional files

Additional file 1: **Figure S1.** In vitro characterization of the immortalized CAF154 fibroblasts. The constitutive high levels of hTERT in all the fibroblasts transduced with retroviral particles (examples shown in A) were confirmed. Nevertheless, almost all the fibroblasts stopped growing after a few population doublings (PDs) and underwent senescence (B) with the exception of CAF154-hTERT cells, which expressed high levels of hTERT (A), showed no signs of senescence (B), and proliferated in a continuous fashion in vitro (C). Cumulative PDs were calculated at the end of every passage in relation to the cell number at the first passage. Of note, despite the immortalization process, CAF154-hTERT maintained the capacity to promote the growth of the adjacent cancer cells in co-culture experiments (D). (TIFF 422 kb)

Additional file 2: **Figure S2.** In vivo characterization of the immortalized CAF154 fibroblasts. To exclude that the ectopic expression of hTERT and the prolonged culturing had affected the capacity of the CAFs to promote tumor engraftment rate, we characterized the pro-tumorigenic properties CAF154-hTERT cells in vivo by co-injecting CAF154-hTERT and A549 cell lines in immunocompromized mice. We found that the ectopic expression of hTERT did not affect the pro-tumorigenic capability of CAFs to promote the tumor take (A), the volume of the subcutaneous nodules (B), and the dissemination of human cells to the lungs (C) compared to the non-transfected counterpart CAF154 cell line. Based on this evidence, we concluded that the immortalization process did not alter the pro-tumorigenic features of CAF154 cells both in vitro and in vivo. (TIFF 166 kb)

Additional file 3: **Figure S3.** Potential miR-16 target regions in HGF, FGFR-1, and MEK1 mRNA. FGFR-1 3′UTR was mutagenized to delete to potential miR-16-directed region. (TIFF 86 kb)

Additional file 4: **Figure S4.** MiR-16 inhibition results in increased HGF levels in primary fibroblasts. Primary fibroblast cell lines were transfected with control miRNA (miR-C inh) and miR-16 inhibitor (miR-16 inh) and CM collected 72 h later (four cell lines in two independent experiments, paired *t* test *p* = 0.0430). (TIFF 78 kb)

Additional file 5: **Figure S5.** FGF-1 and FGF-2 levels are not affected by miR-16. Levels of FGF-1 and FGF-2 in the CM of CAF154-hTERT fibroblasts were transfected with control miR-C and miR-16, collected 72 h after the transfection, and analyzed by multiplex analysis. (TIFF 81 kb)

## Abbreviations

3′UTR: 3′ untranslated region
AF: Adjacent fibroblast
CAF: Cancer-associated fibroblast
CLL: Chronic lymphocytic leukemia
CM: Conditioned medium
EGFR: Epidermal growth factor receptor
ERK1/2: Extracellular signal-regulated kinases 1/2
FGF-2: Fibroblast growth factor-2
FGFR-1: Fibroblast growth factor receptor-1
GFP: Green fluorescent protein
HGF: Hepatocyte growth factor
HTS: High-throughput screening
MEK1/2: Mitogen-activated protein kinase/ERK kinase 1/2
NF: Normal fibroblast
NSCLC: Non-small cell lung cancer
TME: Tumor microenvironment
