# Supplementary figures and images for: MiR-16 regulates the pro-tumorigenic potential of lung fibroblasts through the inhibition of HGF production in an FGFR-1- and MEK1-dependent manner
Source: J Hematol Oncol. 2018 Mar 20;11:45. doi: 10.1186/s13045-018-0594-4 (PMC5861674; doi:10.1186/s13045-018-0594-4)

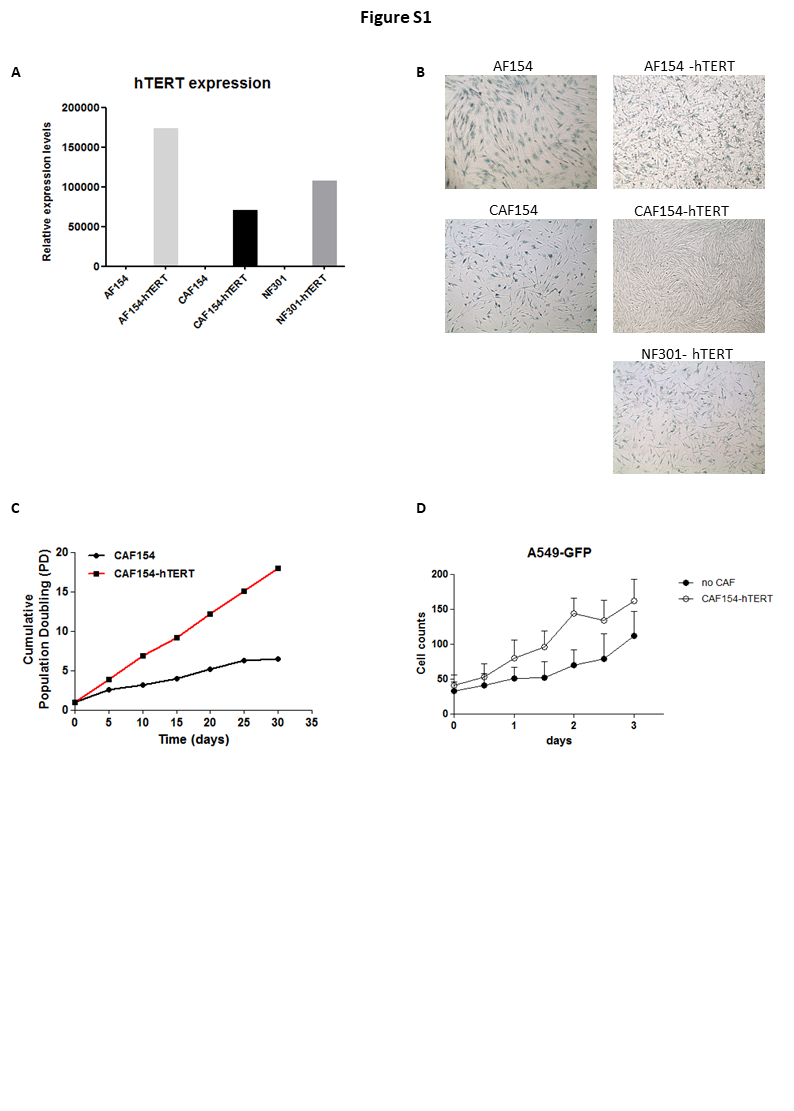

Supplement: Supplementary file 1 — Figure S1. In vitro characterization of the immortalized CAF154 fibroblasts. The constitutive high levels of hTERT in all the fibroblasts transduced with retroviral particles (examples shown in A) were confirmed. Nevertheless, almost all the fibroblasts stopped growing after a few population doublings (PDs) and underwent senescence (B) with the exception of CAF154-hTERT cells, which expressed high levels of hTERT (A), showed no signs of senescence (B), and proliferated in a continuous fashion in vitro (C). Cumulative PDs were calculated at the end of every passage in relation to the cell number at the first passage. Of note, despite the immortalization process, CAF154-hTERT maintained the capacity to promote the growth of the adjacent cancer cells in co-culture experiments (D). (TIFF 422 kb) [file 13045_2018_594_MOESM1_ESM.tif]

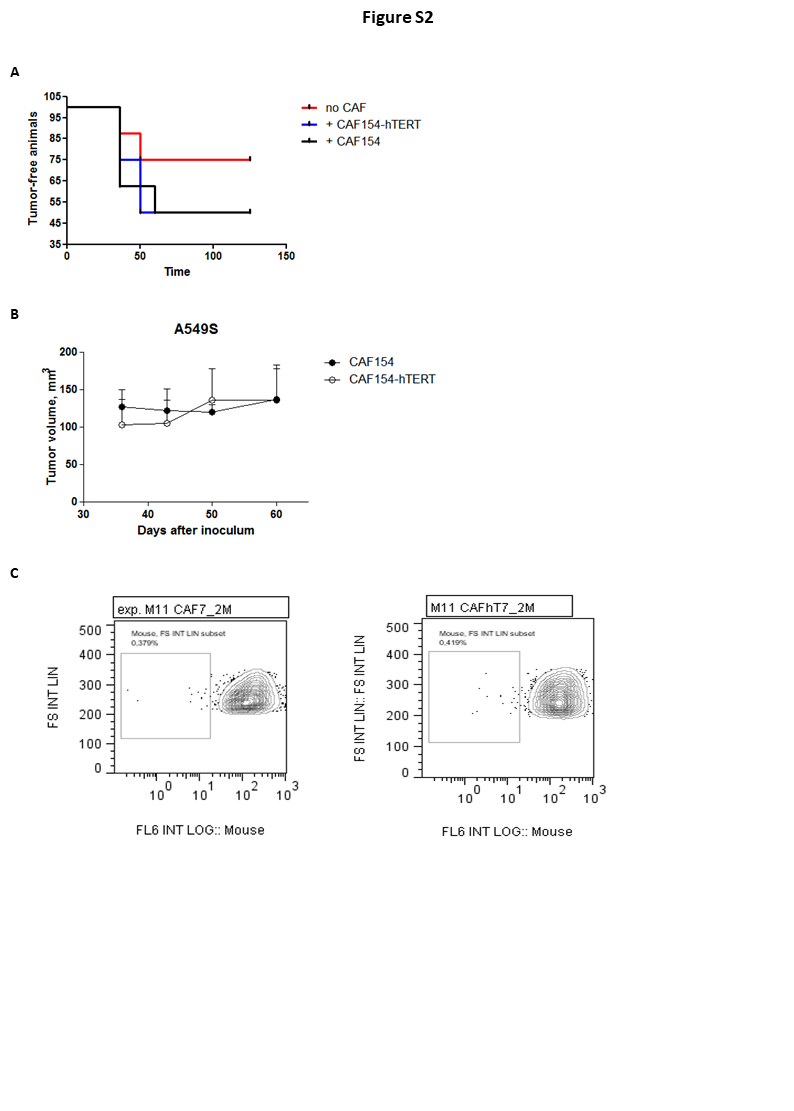

Supplement: Supplementary file 2 — Figure S2. In vivo characterization of the immortalized CAF154 fibroblasts. To exclude that the ectopic expression of hTERT and the prolonged culturing had affected the capacity of the CAFs to promote tumor engraftment rate, we characterized the pro-tumorigenic properties CAF154-hTERT cells in vivo by co-injecting CAF154-hTERT and A549 cell lines in immunocompromized mice. We found that the ectopic expression of hTERT did not affect the pro-tumorigenic capability of CAFs to promote the tumor take (A), the volume of the subcutaneous nodules (B), and the dissemination of human cells to the lungs (C) compared to the non-transfected counterpart CAF154 cell line. Based on this evidence, we concluded that the immortalization process did not alter the pro-tumorigenic features of CAF154 cells both in vitro and in vivo. (TIFF 166 kb) [file 13045_2018_594_MOESM2_ESM.tif]

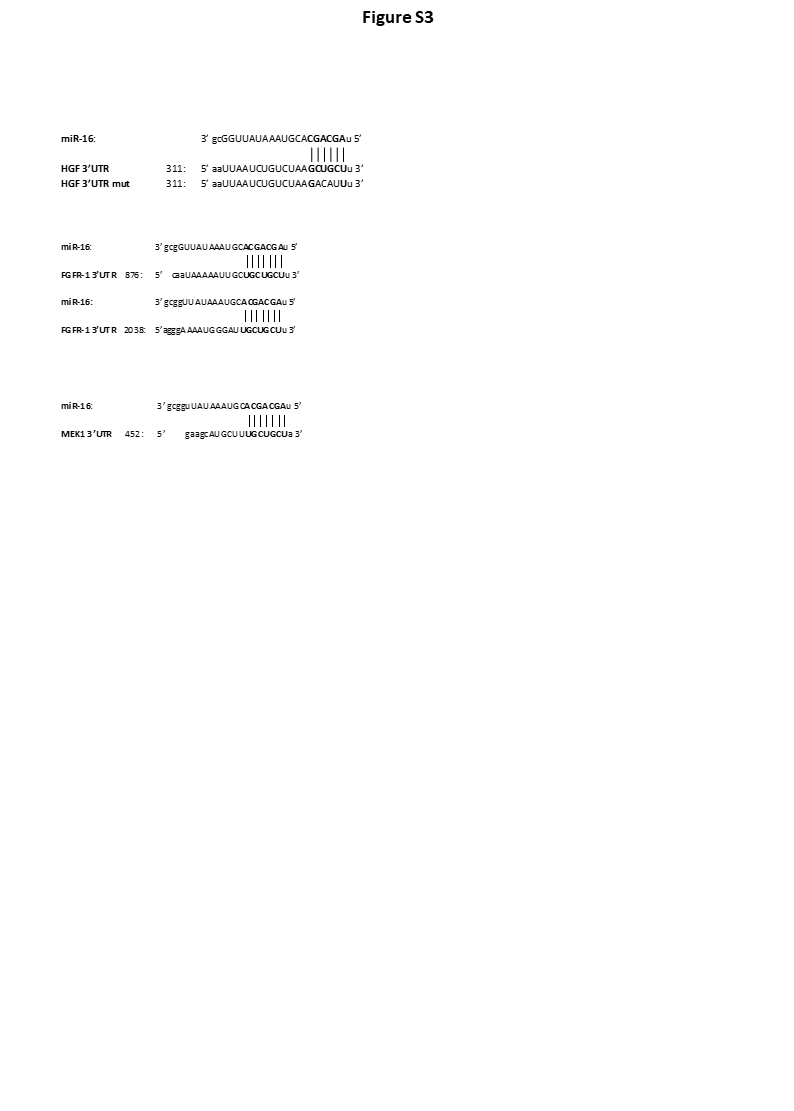

Supplement: Supplementary file 3 — Figure S3. Potential miR-16 target regions in HGF, FGFR-1, and MEK1 mRNA. FGFR-1 3′UTR was mutagenized to delete to potential miR-16-directed region. (TIFF 86 kb) [file 13045_2018_594_MOESM3_ESM.tif]

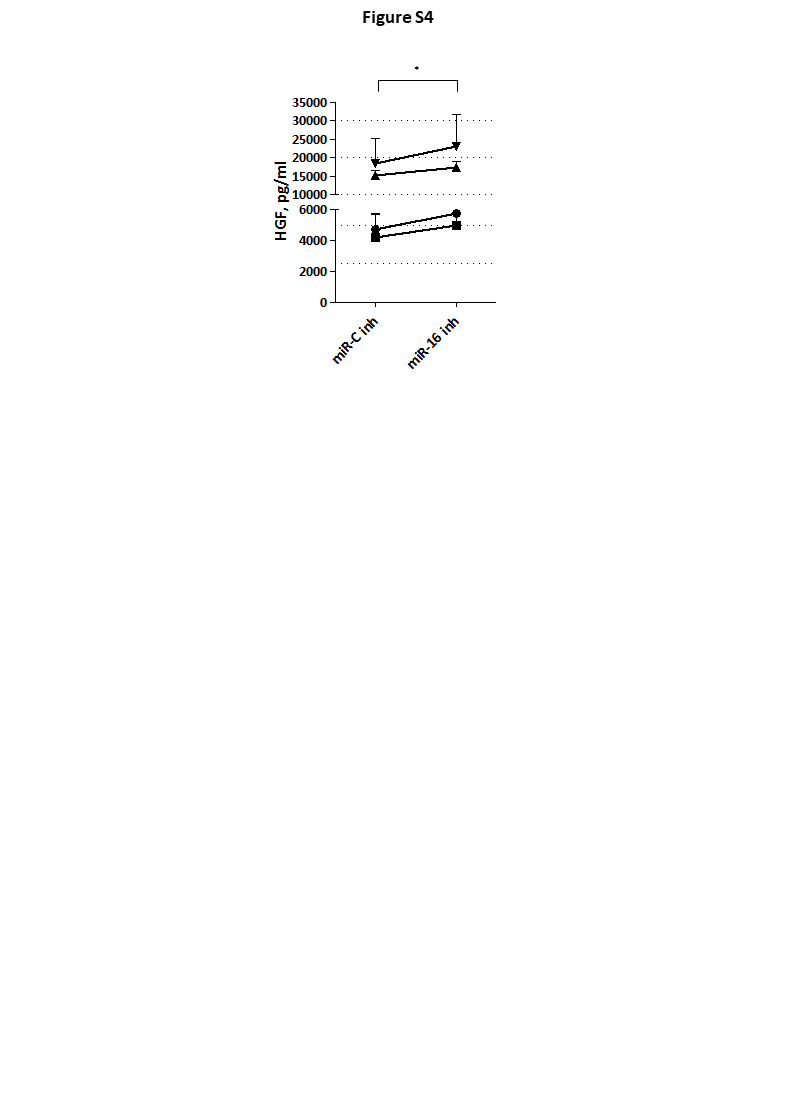

Supplement: Supplementary file 4 — Figure S4. MiR-16 inhibition results in increased HGF levels in primary fibroblasts. Primary fibroblast cell lines were transfected with control miRNA (miR-C inh) and miR-16 inhibitor (miR-16 inh) and CM collected 72 h later (four cell lines in two independent experiments, paired t test p = 0.0430). (TIFF 78 kb) [file 13045_2018_594_MOESM4_ESM.tif]

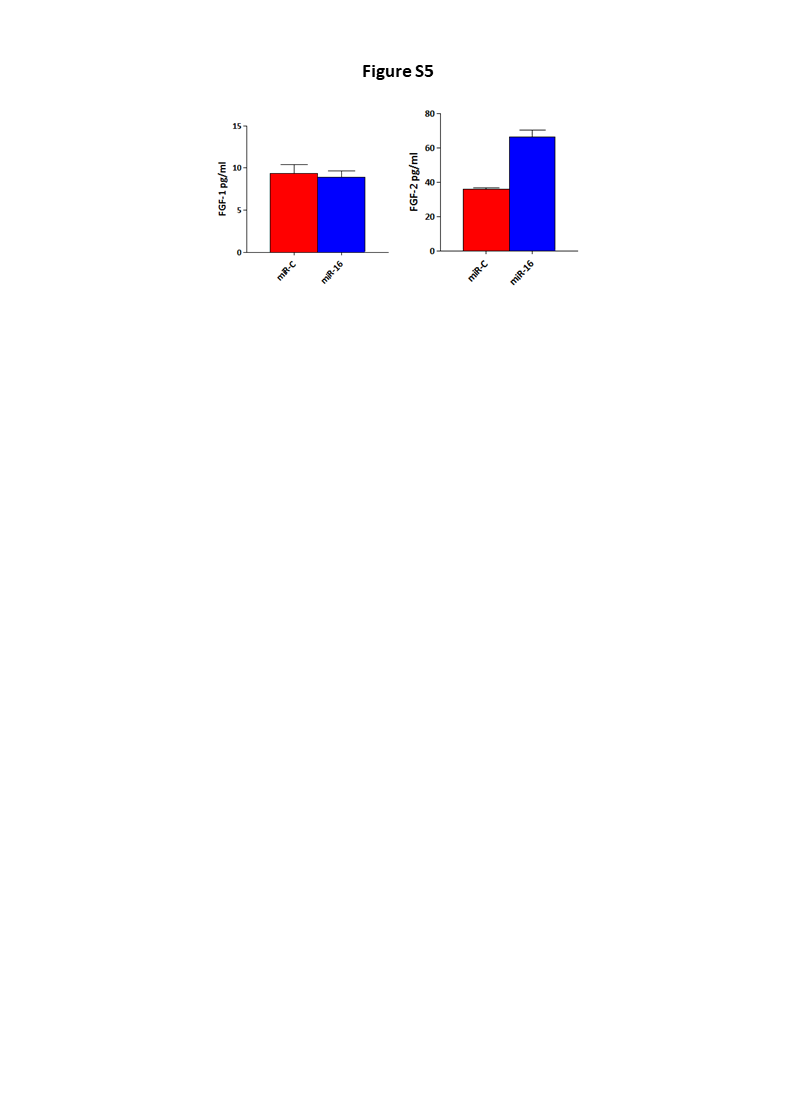

Supplement: Supplementary file 5 — Figure S5. FGF-1 and FGF-2 levels are not affected by miR-16. Levels of FGF-1 and FGF-2 in the CM of CAF154-hTERT fibroblasts were transfected with control miR-C and miR-16, collected 72 h after the transfection, and analyzed by multiplex analysis. (TIFF 81 kb) [file 13045_2018_594_MOESM5_ESM.tif]
